# Iodocionin, a Cytotoxic Iodinated Metabolite from the Mediterranean Ascidian *Ciona edwardsii*

**DOI:** 10.3390/md8020285

**Published:** 2010-02-21

**Authors:** Anna Aiello, Ernesto Fattorusso, Concetta Imperatore, Marialuisa Menna, Werner E.G. Müller

**Affiliations:** 1 Dipartimento di Chimica delle Sostanze Naturali, Università degli Studi di Napoli “Federico II”, Via D. Montesano 49, 80131, Napoli, Italy; E-Mails: aiello@unina.it (A.A.); fattoru@unina.it (E.F.); cimperat@unina.it (C.I.); 2 Dept. for Applied Molecular Biology, Institute for Physiological Chemistry, Johannes Gutenberg- University Medical Center, Duesbergweg 6, D-55099 Mainz, Germany; E-Mail: wmueller@uni-mainz.de

**Keywords:** natural products, iodinated metabolites, cytotoxic activity, structure elucidation

## Abstract

Chemical investigation of the Mediterranean ascidian *Ciona edwardsii* has been performed, leading to the isolation of two halogenated compounds: a new tyrosineiodinated derivative iodocionin (**1**) and the relevant brominated analogue (**2**), previously isolated from a Caribbean sponge. The structure of the new compound **1** has been assigned on the basis of spectroscopic analysis. Both compounds were tested for cytotoxicity *in vitro* against two different cancer cell lines, L5178Y (mouse lymphoma) and PC-12 (rat pheochromocytoma). Iodocionin was shown to possess significant and selective activity against lymphoma cells with an IC_50_ of 7.75 μg/mL.

## 1. Introduction

The sea is a large source of biogenic organohalogens, which are biosynthesized by seaweeds, sponges, corals, tunicates, bacteria, and other marine life [1]. Halogenated tyramine-derivatives frequently occur in marine organisms and are known to play basic functions related to the survival of the living creatures producing them [2]. Bromine is by far the halogen most frequently found in these metabolites; for example, bromotyrosine derivatives are constantly detected among the secondary metabolites of the Demospongiae; thus, they have been used for taxonomic purposes [3–5]. On the contrary, iodinated metabolites, biosynthetically related to tyrosine, are much less widespread in the marine environment, since they have been isolated just in a few algae, microorganisms and marine invertebrates [6,7]. We wish to report here the isolation from a phlebobranchiate ascidian, *Ciona edwardsii* (species similar but distinct from *C. intestinalis*) of a new tyrosine-iodinated derivative, named iodocionin (**1**) together with the relevant brominated analogue **2**, previously isolated from the Caribbean sponge *Verongula gigantea* [8]. Both compounds **1** and **2** were tested for their cytotoxic activity *in vitro* against two different cancer cell lines, L5178Y (mouse lymphoma) and PC-12 (rat pheochromocytoma). Iodocionin (**1**) was shown to possess a significant and selective activity against lymphoma cells.

## 2. Results and Discussion

### 2.1. Isolation and structure elucidation

Specimens of *C. edwardsii* collected in the bay of Naples were exhaustively extracted with methanol and, subsequently, with chloroform. The combined extracts were concentrated *in vacuo*; the resulting residue was partitioned between water and ethyl acetate and the polar layer was re-extracted with butanol. The butanol soluble material was initially subjected to chromatography over reverse phase silica gel column eluting with a solvent gradient from H_2_O/MeOH (9:1) to MeOH 100%. The MeOH/H_2_O 1:1 fraction was further separated by HPLC on a C18 reverse phase column (eluent: MeOH/aqueous TFA 0.1% 7:3), yielding iodocionin (**1**) and compound **2** in the pure state. The structure of the latter compound was deduced by comparison of its spectroscopic properties with those reported in literature [8].

The ESI mass spectrum of iodocionin (**1**) displayed a sole ion peak at *m/z* 306, corresponding to [M]^+^. The molecular formula of **1** was established [HRFAB-MS (positive ions mode) analysis on the peak at *m/z* 306.0362 (calculated value: 306.0349)] as C_11_H_17_INO differing from that of **2** only in having a different halogen atom.

^1^H and ^13^C-NMR features of compound **1** were nearly identical to those of **2**, with regard to the number and shape of the signals. However, differences were observed in the chemical shift values of the signals present in the downfield region of the spectra (see Table 1). Significantly different were the chemical shift values of two aromatic carbons [δ 140.5 in **1** *vs.* δ 134.6 in **2** (C-2′) and δ 84.7 in **1** *vs.* δ 111.2 in **2** (C-3′)] and of one of the aromatic protons [δ 7.68 *vs.* δ 7.49 (H-2′)]. These evidences suggested the presence in **1** of a 1,2,4-trisubstituted benzene ring like in **2** having, as substituents, an hydroxyl, a iodine atom and the -CH_2_CH_2_ ^+^N(CH_3_)_3_ group.

In order to univocally assign the structure of **1**, remained to establish the relative positions of the three substituents [-OH, -I, and -CH_2_CH_2_ ^+^N(CH_3_)_3_]. It was deduced by analysis of HMBC data, as well as trough a quantitative estimation of heteronuclear coupling constants.

**Figure 1:**
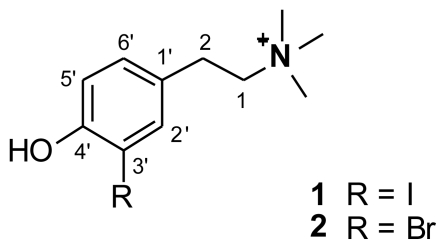
Structures of compounds **1** and **2**.

The methylene protons at δ 3.01 (2H-2) showed key HMBC correlations with the aromatic unprotonated carbon at δ 129.2 (C-1′), as well as with the two methine carbons at δ 130.9 (C-6′) and 140.5 (C-2′), indicating the location of -CH_2_-CH_2_-^+^N(CH_3_)_3_ group at C-1′. Since H-6′ was *ortho* coupled to H-5′, the iodine atom and the hydroxyl group could be attached at C-3′ and C-4′, respectively, or vice versa. The upfield chemical shift value of H-5′ (δ 6.81) was consistent with its location *ortho* to the phenolic group, suggesting for iodocionin the substituents location depicted in Figure 1. However, definitive information on the relative position of the three substituents has been obtained from the quantitative evaluation of ^n^*J*(C,H) coupling constants (Table 2) obtained through inspection of phase-sensitive HMBC data. The above evaluation could be easily accomplished since the carbons linked to the OH (δ 157.3) and I (δ 84.7), resonating at very different chemical shifts, were unambiguously identified. In detail, H-2′ (δ 7.68) showed typical ^3^*J*_C-H_ large couplings with C-4′ (δ 157.3) and C-6′ (δ 130.9), while a small coupling was found with C-3′ (δ 84.7) [9]. Accordingly, H-5′ (δ 6.81) showed large ^3^*J*_C-H_ coupling constants with C-1′ (δ 129.2) and C-3′, and small coupling constants with C-2′ (δ 140.5) and C-4′. Furthermore, H-6′ (δ 7.14) exhibited large coupling constants with C-2′ and C-4′, while small coupling constants with C-3′ and C-5′ (δ 115.7). The above results univocally indicated the relative position of each substituent on the aromatic nucleus, thus defining the structure of iodocionin as 2-(4-hydroxy-3-iodophenyl)-*N,N,N*-trimethylethanaminium.

### 2.2. Biological activities of compounds 1 and 2

**Figure 2:**
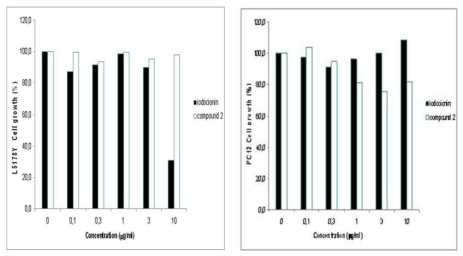
Effect of compounds **1** and **2** on L5178Y and PC-12 cell lines.

The capability of both compounds **1** and **2** to affect cell viability has been estimated *in vitro* on two different cell lines, L5178Y (mouse lymphoma) and PC12 (rat pheochromocytoma), using the microculture tetrazolium (MTT) assay. The results were statistically evaluated using the paired Student’s *t*-test [10]. Iodocionin (**1**) exhibited an acute activity against lymphoma cells with an IC_50_ of 7.75 μg/mL, showing 70% inhibition of cell growth at a concentration of 10 μg/mL, while it did not affect at all the viability of PC12 cells. In contrast, compound **2** displayed in both cell systems no statistically significant reduction of cell growth at concentrations between 0.1 and 10 μg/mL. These preliminary results, illustrated in Figure 2, indicate that iodocionin seems to selectively inhibit lymphoma rather than pheochromocytoma cell proliferation. Moreover, comparison of the effects of compounds **1** and **2** revealed a definite structure–activity relationship for the nature of the halogen atom present on the aromatic ring.

## 3. Experimental Section

### 3.1. General Experimental Procedures

ESI mass spectra were obtained on an API 200 mass spectrometer. HRFABMS (glycerol matrix) were performed on a VG Prospec (FISONS) mass spectrometer. NMR experiments were performed on a Varian Unity INOVA 500 spectrometer; chemical shifts are referred to the residual solvent signal (CD_3_OD: δ_H_ = 3.31, δ_C_ = 49.0). Medium-pressure liquid chromatographies (MPLC) were carried out on a Buchi 861 apparatus with SiO_2_ (230–400 mesh) packed columns. High-performance liquid chromatography (HPLC) separations were achieved on a Waters 501 apparatus equipped with an RI detector.

### 3.2. Extraction and Isolation

Specimens of *C. edwardsii* were collected at −75 m depth in the autumn of 2006 in the bay of Naples (Meta di Sorrento, Punta Gradelle) and kept frozen until used. A voucher specimen was deposited at the Dipartimento di Chimica delle Sostanze Naturali, Napoli, Italy. The freshly thawed tunicate (21.9 g dry weight after extraction) was homogenized and treated at room temperature with methanol (3 × 1 L) and, subsequently, with chloroform (3 × 1 L). The combined extracts were concentrated *in vacuo* to give an aqueous suspension that was subsequently extracted initially with EtOAc and then with BuOH. The butanol soluble material (5.4 g of a dark brown oil), obtained after evaporation of the solvent, was chromatographed on a RP-18 silica gel flash column using a gradient elution (water → methanol → chloroform).

The fractions eluted with H_2_O/MeOH 1:1 were chromatographed by HPLC on an RP-18 column (Luna, 3 μm, 150 × 4.60 mm), using MeOH/H_2_O 7:3 containing TFA 0.1% as the eluent (flow 0.5 mL/min). This separation afforded 4.5 mg of pure iodocionin (**1**) and 1.5 mg of compound **2**, identified by comparison of its spectral properties with literature values.

#### Iodocionin (1)

Yellow viscous oil; ESI-MS (positive ion mode): *m/z* = 306 [M]^+^; HRFABMS (positive ion mode): *m/z* = 306.0362; the molecular formula C_11_H_17_INO requires 306.0349; ^1^H NMR and ^13^C NMR data (CD_3_OD, 500 MHz) are reported in Table 1.

### 3.3. Cytotoxicity Assays

The cytotoxicity against L5178Y (mouse lymphoma) and PC-12 (rat pheochromocytoma) cells was determined using the microculture tetrazolium (MTT) assay and compared to that of untreated controls [11–13]. Stock solutions of the test samples in EtOH 96% (v/v) were prepared. Exponentially growing cells were harvested, counted, and diluted to the appropriate concentration. 50 μL of the cell suspension, containing 3750 cells, were pipetted into 96-well microtiter plates; subsequently, 50 μL of a solution of the test samples, containing the appropriate concentration, were added to each well. A concentration range between 0.1 and 10 μg/mL was chosen. As established in a preliminary experiment, the low amounts of EtOH used in the assays did not affect the cells growth. The plates were incubated at 37 °C under 5% CO_2_ atmosphere for 72 h. Then, a stock solution of 3-(4,5-dimethylthiazol-2-yl)-2,5-diphenyltetrazolium bromide (MTT; Sigma) was prepared (5 mg/mL in phosphate-buffered saline (PBS; 1.5 mM KH_2_PO_4_, 6.5 mM Na_2_HPO_4_, 137 mM NaCl, 2.7 mM KCl; pH 7.4); then, 20 μL of this solution were pipetted into each well. The yellow-colored MTT penetrates into living cells and there undergoes enzymatic conversion to a blue formazan complex due to mitochondrial dehydrogenases. After an incubation period of 4 h at 37 °C in a humidified 5% CO_2_ incubator, the medium was centrifuged (15 min; 20 °C; 210 g), cells lysed with dimethyl sulfoxide, and the absorbance was measured at 520 nm using a scanning microtiter-well spectrophotometer. The color intensity is correlated with the number of healthy living cells; thus, cell survival was calculated using the formula:

survival (%)=[100 × (absorbance of treated cells - absorbance of culture medium)]:[(absorbance of untreated cells - absorbance of culture medium)].

All experiments were carried out in ten parallel assays and repeated three times. As controls, media with 0.1% EGMME/DMSO were included in the experiments, as previously described [14].

## 4. Conclusions

The number of halometabolites isolated from natural sources and structurally defined continues to increase, but most of the biogenic organohalogens discovered over the past 35 years are marinederived, biosynthesized by almost all the marine life [1]. It has been suggested that marine organisms might have indeed a vested interest in the biosynthesis of halogenated compounds since halogenation of organic molecules often confers high levels of biological activity even in precursors with little or no intrinsic activity [15]. Thus, the potential usefulness of these compounds for human therapy could be a driving force for their continuing pursuit.

## Figures and Tables

**Table 1:** NMR data (CD_3_OD) of 1 and 2.

	1		2	

Pos.	δ_H_ (mult., *J* in Hz)	δ_C_	δ_H_ (mult., *J* in Hz)	δ_C_
**1′**	-	129.2	-	129.2
**2′**	7.68 (d, 2.1)	140.5	7.49 (d, 1.5)	134.6
**3′**	-	84.7	-	111.2
**4′**	-	157.3	-	154.9
**5′**	6.81 (d, 8.3)	115.7	6.90 (d, 8.1)	117.7
**6′**	7.14 (dd, 8.3, 2.1)	130.9	7.15 (dd, 8.1, 1.5)	130.3
**1**	3.50 (m)	68.2	3.53 (m)	68.5
**2**	3.01 (m)	28.6	3.05 (m)	29.1
***N*(CH_3_)_3_**	3.19 (s)	53.4	3.22 (s)	53.7

**Table 2:** Heteronuclear coupling constant pattern for **1**.

	C-1′	C-2′	C-3′	C-4′	C-5′	C-6′
**H-2′**	-	-	3.8 Hz	8.7 Hz	-	9.0 Hz
**H-5′**	7.5 Hz	−1.0 Hz	7.5 Hz	4.4 Hz	-	-
**H-6′**	-	7.4 Hz	−1.4 Hz	7.4 Hz	1.0 Hz	-
